# Differential responses of contrasting low phosphorus tolerant cotton genotypes under low phosphorus and drought stress

**DOI:** 10.1186/s12870-023-04171-5

**Published:** 2023-03-30

**Authors:** Asif Iqbal, Gui Huiping, Dong Qiang, Wang Xiangru, Zhang Hengheng, Zhang Xiling, Song Meizhen

**Affiliations:** 1 State Key Laboratory of Cotton Biology, Zhengzhou Research Base, School of Agricultural Sciences, State Key Laboratory of Cotton Biology, Institute of Cotton Research of Chinese Academy of Agricultural Sciences, Zhengzhou University, Anyang, Henan 455000 People’s Republic of China; 2 Western Agricultural Research Center of Chinese Academy of Agricultural Sciences, Changji, Xinjiang 831100 China; 3 Department of Agriculture, Hazara University, Khyber Pakhtunkhwa, Mansehra, 21120 Pakistan

**Keywords:** Cotton, Phosphorus, Drought stress, Photosynthesis, Antioxidant system, osmotic adjustment

## Abstract

**Background:**

Drought is one of the main reasons for low phosphorus (P) solubility and availability.

**Aims:**

The use of low P tolerant cotton genotypes might be a possible option to grow in drought conditions.

**Methods:**

This study investigates the tolerance to drought stress in contrasting low P-tolerant cotton genotypes (Jimian169; strong tolerant to low P and DES926; weak tolerant to low P). In hydroponic culture, the drought was artificially induced with 10% PEG in both cotton genotypes followed by low (0.01 mM KH_2_PO_4_) and normal (1 mM KH_2_PO_4_) P application.

**Results:**

The results showed that under low P, PEG-induced drought greatly inhibited growth, dry matter production, photosynthesis, P use efficiency, and led to oxidative stress from excessive malondialdehyde (MDA) and higher accumulation of reactive oxygen species (ROS), and these effects were more in DES926 than Jimian169. Moreover, Jimian169 alleviated oxidative damage by improving the antioxidant system, photosynthetic activities, and an increase in the levels of osmoprotectants like free amino acids, total soluble proteins, total soluble sugars, and proline.

**Conclusions:**

The present study suggests that the low P-tolerant cotton genotype can tolerate drought conditions through high photosynthesis, antioxidant capacity, and osmotic adjustment.

**Supplementary Information:**

The online version contains supplementary material available at 10.1186/s12870-023-04171-5.

## Introduction

Drought is one of the most important abiotic stresses [1] that inhibits the growth and development of plant and affect the balance of water flux, stomatal closure, and carbon dioxide (CO_2_) fixation [2]. Drought badly affects several plant physiological processes like photosynthesis [3] and carbon metabolism [4]. The sensitivity of plants against drought is shown by the reduction in chlorophyll, photosynthesis, enzymatic activities, and gene expression which resulted in poor growth and production [5, 6]. The long-term drought condition inhibited photosynthetic CO_2_ assimilation through stomatal and non-stomatal factors [7]. The nonstomatal factors consisted of a reduction in linear electron transport, actual quantum yield, and maximum quantum yield of electron flow through photosystem II [8]). These reductions are related to the downregulation of light reaction processes like increasing nonphotochemical quenching [9]. Moreover, inhibition of CO_2_ resulted in more excitation energy and electron fluxes to O_2_, which leads to photo-oxidative damage of the cell component through an excess generation of reactive oxygen species (ROS), and ultimately photoinhibition [10]. The ROS consisting of superoxide (O_2_^−^) and hydrogen peroxides (H_2_O_2_), form natural products in oxygen metabolism and work as secondary messengers in redox signal transduction. However, excessive production of ROS under drought stress leads to oxidative damage of proteins, DNA, and lipids that subsequently inhibit plant growth [11].

Plants have developed several protective mechanisms to protect the photosynthetic apparatus from the damage caused by ROS and photoinhibition. As variation in the size of the light-harvesting antenna may reduce the absorption of light energy when the CO_2_ assimilation ability is lower [12]. Additionally, photochemical and non-photochemical pathways are associated with avoiding the excess excitation energy from the electron transport chain of photosynthesis [13]. Moreover, ROS-scavenging antioxidant enzymes like superoxide dismutase (SOD), peroxidase (POD), catalase (CAT), and non-enzymatic antioxidant systems like glutathione, ascorbate, and carotenoids are responsible for managing excessive energy to overcome the oxidative stress [14]. These enzymes can directly scavenge ROS or produce non-enzymatic antioxidants. Among them, SOD is responsible for the dismutation of O_2_^−^ into H_2_O_2_ in various cell components, while POD scavenges H_2_O_2_ produced from O_2_^−^ produced via SOD dismutation. Moreover, CAT eliminates H_2_O_2_ in the mitochondria and microbody thereby ameliorating the negative impacts of oxidative stress [15].

Another way to avoid cellular damage from dehydration and maintain normal growth under drought is to reduce the osmotic potential. Through this strategy, the water enters the cells via a potential gradient and the plants accumulate more solutes to reduce the osmotic potential. These solutes play a key role in maintaining the osmotic equilibrium and protecting the macromolecules and membranes, thereby improving the resistance against drought stress and cellular dehydration [11]. Particularly, the hydroxyl group of sugar alcohols replaces the OH group of the water to sustain the hydrophilic connections with the membrane proteins and lipids. Therefore, these solutes help the plants to maintain the strength and function of the membranes without affecting the normal metabolism of the cell. The accumulation of these solutes improves the functional abilities of the plants under stress conditions, however, the capabilities depend on plant species, cultivars, plant tissues, stress intensity, and the growth stage [16]. Currently, the mechanism of drought stress tolerance and osmotic adjustment to sustain metabolic function in cotton is to be elucidated.

Drought stress reduced plant growth not only through the inhibition of stomatal conductance and photosynthesis but also by reducing nutrient uptake, transport, and assimilation [17]. Phosphorus (P) is one of the major nutrients required for plant growth. However, its low mobility in the soil causes its deficiency and as a result, several morphological, physiological, and biochemical variations occur in plants [18, 19]. Generally, the availability of P is low due to precipitation and the rate of absorption in the rhizosphere exceeds the rate of its replenishment in soil solution [20]. Drought restricts P uptake by reducing the availability of P in the rhizosphere along with various other factors that affect plant water relations [21]. Previous reports suggested that P is responsible for the development of an extensive root system and that its deficiency exacerbates drought stress [22, 23]. The application of P fertilizer can reduce P deficiency, improve the plant’s stress tolerance [24] and finally adjust the morphological, physiological, and biochemical processes that increase plant growth [25–27]. Regardless of P importance for plant productivity, few studies have investigated the impact of P on plant physiological processes under drought stress [26, 27].

Cotton is the leading fiber crop grown throughout the world, providing raw materials to the textile industry [28, 29]. In China, most of the cotton is shifted from the Yellow River and Yangtze River valley to Xinjiang province [30]. However, Xinjiang is an arid region having low precipitation and high surface evaporation and are therefore facing the issues like scarcity of water resources [31] and low nutrient availability, especially P [32]. Thus, improving the ability of cotton to tolerate drought stress will become a major scientific issue in the future. We hypothesized that the application of P and the use of low-P tolerant genotypes can improve drought stress tolerance by inducing plant morphology, photosynthetic efficiency, osmotic adjustment, and antioxidant enzymatic activities in cotton. Therefore, the study aims to know the morphological and physiological responses of contrasting low P-tolerant cotton genotypes and the alleviation of the adverse effects of drought by enhancing the drought tolerance potential under low and normal P conditions.

## Materials and Methods

### Plant growth conditions and experimental design

A hydroponic experiment was conducted in the greenhouse at the Cotton Research Institute of the Chinese Academy of Agricultural Sciences (CRI, CAAS), Anyang, China. According to the previous study, two cotton genotypes Jimian169 (strong tolerance to low P) and DES926 (weak tolerance to low P) were used in the experiment [33]. The seeds of selected contrasting low P-tolerant cotton genotypes were kindly provided by the CRI, CAAS, China. The selected seed permission was granted from the respective authority. Healthy and uniform seeds of both cotton genotypes were sown in sterilized sand in an incubator for one week. After germination, uniform healthy plants were transplanted into a plastic container (7 L) in a growth condition of 16/8 h light/dark cycle, 28 °C temperature, and 60% relative humidity. During the first week, ½ Hoagland solution was applied followed by a full-strength till end of the experiment [18]. After the development of three true leaves, plants of both cotton genotypes were divided into four groups: (i) low P with drought stress (LP + DS; 0.01 mM KH_2_PO_4_ + 10% PEG); (ii) low P without drought stress (LP + CK; 0.01 mM KH_2_PO_4_ + 0% PEG); (iii) normal P with drought stress (NP + DS; 1 mM KH_2_PO_4_ + 10% PEG); (iv) normal P without drought stress (NP + DSCK; 1 mM KH_2_PO_4_ + 0% PEG). The seedlings were aerated with an electric pump and the solutions were renewed once a week. To avoid the edge effects, the position of the boxes was interchanged when refreshing the solutions. After two weeks of treatment, with obvious morphological variation, the seedlings of both cotton genotypes were harvested and various morphophysiological traits were measured.

### Plant morphology

From each treatment, six plants were randomly selected and the shoot length was measured with the help of calibrated scale [34]. After harvesting, the plants were divided into roots and shoots and subsequently dried at 105 and 80 °C for one and 48 h, respectively. After complete drying, the shoot, root, and total dry matter were determined using an electric balance. At the same time, the roots of half of the plants from each genotype were scanned and analyzed through WinRHIZO root analyzer system [35].

### Measurements of leaf physiological traits

The photosynthetic traits like net photosynthesis, stomatal conductance, transpiration rate, and intercellular CO_2_ concentration were measured from the third fully expanded leaf by using the photosynthetic machine (Li-Cor 6800, USA) from 9:00 to 11:00 a.m. [36]. About 50 mg of fresh leaf sample was used to measure chlorophyll and carotenoid contents. The collected samples were crushed and kept overnight in acetone: ethanol (1:1) solution for 48 h at 25 °C. The absorbance values for chlorophyll and carotenoid contents were measured according to our previous study [37].

### Determination of phosphorus concentration and use efficiency

P concentration in root and shoot tissues were measured according to the Kjeldahl method [38]. The grounded sample of 0.2 g from each tissue was digested with H_2_SO_4_-H_2_O_2_, and the final P concentration was analyzed with the help of Continous Flow Auto Analyzer III. The various P-use efficiency-related traits were measured according to our previous study [18].

### Determination of malonaldehyde contents and reactive oxygen species

The malonaldehyde (MDA) content in root and shoot was measured by the thiobarbituric acid (TBA) reactions according to the standard protocol [39]. The samples (0.2 g) were extracted in 2 ml of 0.25% TBA prepared in 10% TCA. The extract was heated at 95°C for 30 min, and then, quickly cooled on ice. The collected extract was centrifuged at 10,000 g for 10 min and absorbance was measured at 532 nm.

The H_2_O_2_ content was determined by using the previously published method [40]. The root and shoot samples (0.1 g) were crushed and homogenized with 5 ml of 0.1% TCA and centrifuged at 12,000 g for 15 min. After centrifugation, about 0.5 ml of supernatant was collected and 1 ml of 1 M potassium iodide and 0.5 ml of 10 mM phosphate buffer were added. Finally, H_2_O_2_ content was measured at 390 nm using a spectrophotometer.

The O_2_^−^ content was measured according to the previously published protocol [40]. An amount of 0.2 g of root and shoot samples was homogenized with 1 ml of BPS 65 mM (pH 7.8) followed by centrifugation at 10,000 g for 10 min. The supernatant was collected in a new tube and subsequently, 75 µL of BPS and 25 µL of 10 mM hydroxylamine hydrochloride were added and then incubated for 20 min at room temperature. Further, 1 ml of supernatant was taken and combined with 1 ml of 7 mM α-naphthalene diamine hydrochloride and 1 mL of 17 mM a-sulphanilamide. Finally, 3 ml of ether was added and centrifuged at 5000 g under room temperature. The absorbance was measured at 540 nm using a spectrophotometer.

### Determination of antioxidant enzymatic activities

For measuring the enzymatic activities, about 0.5 g of root and shoot samples were crushed in liquid nitrogen and 10 ml of 50 mM sodium phosphate buffer consisting of 1% polyvinyl pyrrolidine, 0.2 mmol·L^− 1^ ethylenediamine tetraacetic acid, and 10 mmol·L^− 1^ magnesium chloride was added. The solution was then centrifuged at 12,000 g for 12 min at 4 °C. Finally, the collected supernatant was stored at 4 °C and the POD activity was measured according to the protocol mentioned in the earlier study [37].

SOD activity was assayed using the photochemical NBT method. The assay mix (1 ml) contained 50 mM phosphate buffer (pH 7.8), 9.9 mM methionine, 57 mM NBT, 0.025% Triton X-100, and 0.0044% riboflavin. The photoreduction of NBT was measured at 560 nm. One unit of SOD was defined as the volume of extract that causes inhibition of the photoreduction of NBT by 50%.

Catalase activity was determined in the homogenates by measuring the decrease in absorption at 240 nm as H_2_O_2_ and enzyme activity expressed as µmol H_2_O_2_ oxidized min^− 1^ g^− 1^ protein. In this case, 50 µl enzyme extract was added to a mixture that contained 50 mM sodium phosphate buffer (pH 7.0) and 10 mM H_2_O_2_ to make the volume 3 ml. Catalase activity was calculated by using an extinction coefficient of 39.4 mM^− 1^ cm^− 1^.

Finally, the collected supernatant was stored at 4 °C, and the SOD, POD, and CAT activities were measured according to the protocol mentioned in earlier studies [36].

### Measurement of osmoprotectants

A previously developed protocol [41] with little modifications was used for the measurement of free amino acids [42]. Acetic acid/sodium acetate (pH 5.4) was used as an extraction buffer and the free amino acid values were measured with a spectrophotometer at 580 nm.

The total soluble proteins were measured according to the previous method [43], using albumin bovine [44]. The root and shoot samples (0.5 g) were crushed in a 5 ml phosphate buffer. The extract was kept in a water bath for 10 min at 100 °C followed by centrifugation at room temperature for 5 min at 5000 g. The reaction mixture consisted of 2 ml dH_2_O, 20 µl enzyme extract, and 0.5 ml Bradford reagent were used. The final values for total soluble protein were recorded at 595 nm using a spectrophotometer.

Total soluble sugars were measured according to the standard protocol with little modifications [45]. About 0.5 g root and shoot samples were homogenized in 3 ml 90% ethanol followed by incubation at 70 °C. Further, 90% ethanol was again added to make the volume up to 25 ml. About 1 ml of supernatant was collected and mixed with anthrone solution and sulfuric acid each of 5 ml. Finally, the values for total soluble sug-ars were recorded at 485 nm using glucose as the standard.

The proline content was determined by weighing a 0.1 g sample and homogenizing it in a 1 mL sulfosalicylic acid solution. After homogenization, the homogenate was kept in a water bath at 95 °C for 10 min and well shaken. After incubation, the homogenates were then centrifuged at 25 °C for 10 min at 10,000 rpm. The supernatant was collected and kept on ice for further use. Next, 0.25 mL sample, 0.25 mL glacial acetic acid, and 0.25 mL ninhydrin, glacial acetic acid, and concentrated phosphoric acid solution were placed into a tube and kept in a water bath for 30 min at 95 °C and shaken well every 10 min. Then, the mixture was cooled to room temperature. Overall, 0.5 mL toluene was added and shaken for 30 s, then left to stand for a while. About 0.2 mL solution was taken from the upper portion, and the absorbance was recorded at 520 nm.

### Statistical analysis

The data were arranged in excel and analyzed with Statistix 10 software using two-way ANOVA with split plot arrangement. The combinations of P and drought stress were considered as the main plot, whereas cotton genotypes were used as a subplot factor. The least significant difference test was used to separate the mean at a probability of 5%. Correlation analysis was performed in OriginPro (2018). All the figures expressed as mean ± standard error of three technical and biological replications were drawn in Graphpad Prism 8.

## Results

### Plant morphology

At the end of the experiment, clear morphological differences were observed in cotton genotypes under low and normal P conditions in drought as well as in control. To evaluate, these changes, various morphological and physiological traits were measured. In comparison with control, drought stress reduced shoot length, root dry matter, shoot dry matter, and total plant dry matter by 13.0%, 25.8%, 39.5%, and 37.0% under low P, while 19.9%, 23.4%, 36.8%, and 34.7% under normal P conditions (Table 1). Irrespective of the treatments, Jimian169 has significantly higher shoot length (11.4%), root dry matter (15.8%), shoot dry matter (17.2%), and total plant dry matter (17.0%) as compared to DES926 (Table 1). Moreover, drought stress decreased root morphological traits like root length (3.9% and 16.2%), root surface area (20.7% and 17.0%), root diameter (21.1% and 18.0%), and root volume (19.8% and 16.8%) under both low and normal P conditions (Table 1). However, the reduction was more in DES926, where Jimian169 had significantly higher root length (9.6%), root surface area (9.5%), root diameter (16.6%), and root volume (10.6%) under both low and normal P conditions (Table 1).

**Table 1:** Shoot length (SL; cm), root dry matter (RDM; g plant^− 1^), shoot dry matter (SDM; g plant^− 1^), total plant dry matter (TDM; g plant^− 1^), root length (RL; m), root surface area (RSA; cm^2^), root diameter (RD; mm), and root volume (RV; cm^3^), of Jimian169 and DES926 under LP + DS (0.01 mM KH_2_PO_4_ + 10%PEG), LP + CK (0.01 mM KH_2_PO_4_ + 0%PEG), NP + DS (1 mM KH_2_PO_4_ + 10%PEG), NP + CK (1 mM KH_2_PO_4_ + 0%PEG).

Genotypes	Treatments	SL	RDM	SDM	TDM	RL	RSA	RD	RV
DES926	LP + DS	9.00 f	0.15 e	0.42 e	0.57 e	12.57 bcd	184 d	0.41 e	2.63 de
	LP + CK	10.67 ef	0.22 d	0.92 d	1.14 d	9.80 d	223 b	0.46 d	3.04 c
	NP + DS	12.93 cd	0.26 c	1.19 c	1.45 c	13.22 bc	168 f	0.46 d	2.41 f
	NP + CK	16.10 b	0.32 b	1.81 b	2.13 b	14.99 ab	190 c	0.52 c	2.71 d
Jimian169	LP + DS	10.43 ef	0.19 d	0.79 d	0.98 d	10.70 cd	192 c	0.44 d	2.71 d
	LP + CK	11.67 de	0.24 c	1.09 c	1.33 c	14.40 ab	251 a	0.62 b	3.60 a
	NP + DS	14.60 bc	0.29 b	1.27 c	1.57 c	13.71 bc	177 e	0.50 c	2.53 ef
	NP + CK	18.27 a	0.40 a	2.09 a	2.49 a	17.16 a	226 b	0.66 a	3.22 b
Genotypes (G)		***	***	***	***	ns	***	***	***
Treatments (T)		***	***	***	***	*	***	***	***
G x T		ns	*	*	*	ns	***	***	***

Note: Means followed by the same letters within the same category in the same columns are not different statistically. * Significant at P ≤ 0.05. ** Significant at P ≤ 0.01. ns = non-significant at P ≥ 0.05. LSD; least significant difference

### Leaf physiology

Except intercellular CO_2_ concentration, drought stress greatly reduced the leaf’s physiological traits like photosynthetic rate (30.5% and 15.9%), stomatal conductance (8.5% and 11.9%), transpiration rate (2.4% and 1.8%), chlorophyll a content (5.9% and 26.2%), chlorophyll b content (11.5% and 28.1%), total chlorophyll content (7.0% and 26.6%), and carotenoid content (4.4% and 4.3%) of cotton genotypes under both low and normal P conditions (Table 2). Irrespective of the treatments, photosynthetic rate, stomatal conductance, transpiration rate, chlorophyll a, chlorophyll b, total chlorophyll, and carotenoid contents s of Jimian169 were higher by 16.3%, 8.0%, 26.7%, 14.2%, 12.0%, 13.8%, and 0.9% than DES926, respectively (Table 2). Under drought stress, comparatively higher values of intercellular CO_2_ concentration for DES926 suggested poor carboxylating efficiencies for the available carbon dioxide.

**Table 2:** Photosynthetic rate (A; µmol m^− 2^ s^− 1^), stomatal conductance (gs; mmol H_2_O m^− 2^ s^− 1^), transpiration rate (E; mmol m^− 2^ s^− 1^), intercellular CO_2_ concentration (Ci; µmol CO_2_ mol^− 1^ air), chlorophyll a (Chl a; mg g^− 1^), chlorophyll b (Chl b; mg g^− 1^), total chlorophyll (Chl a + b; mg g^− 1^), and carotenoid contents (Car; mg g^− 1^), of Jimian169 and DES926 under LP + DS (0.01 mM KH_2_PO_4_ + 10%PEG), LP + CK (0.01 mM KH_2_PO_4_ + 0%PEG), NP + DS (1 mM KH_2_PO_4_ + 10%PEG), NP + CK (1 mM KH_2_PO_4_ + 0%PEG).

Genotypes	Treatments	Pn	gs	E	Ci	Chl a	Chl b	Chl a + b	Car
DES926	LP + DS	3.98 e	0.23 f	2.45 c	329	3.90 f	0.91 f	4.81 f	2.10 d
	LP + CK	6.76 d	0.26 e	2.53 c	307	4.16 ef	1.06 e	5.22 ef	2.20 bc
	NP + DS	7.67 c	0.30 c	3.73 b	294	5.35 cd	1.35 d	6.69 c	2.17 c
	NP + CK	9.22 b	0.35 b	3.81 b	236	6.94 b	1.80 b	8.74 b	2.27 a
Jimian169	LP + DS	6.03 d	0.26 ed	3.76 b	301	4.61 ef	1.00 e	5.61 de	2.13 d
	LP + CK	7.65 c	0.28 cd	3.84 b	273	4.88 de	1.10 e	5.98 d	2.23 b
	NP + DS	8.88 b	0.33 b	4.71 a	259	5.91 c	1.52 c	7.43 c	2.19 bc
	NP + CK	10.46 a	0.37 a	4.79 a	212	8.32 a	2.19 a	10.50 a	2.28 a
Genotypes (G)		***	***	***	***	***	***	***	***
Treatments (T)		***	***	***	***	***	***	***	*
G x T		ns	ns	ns	ns	ns	***	ns	ns

Note: Means followed by the same letters within the same category in the same columns are not different statistically. * Significant at P ≤ 0.05. ** Significant at P ≤ 0.01. *** Significant at P ≤ 0.001. ns = non-significant at P ≥ 0.05. LSD; least significant difference

### Phosphorus concentration and use efficiency

**Fig. 1:**
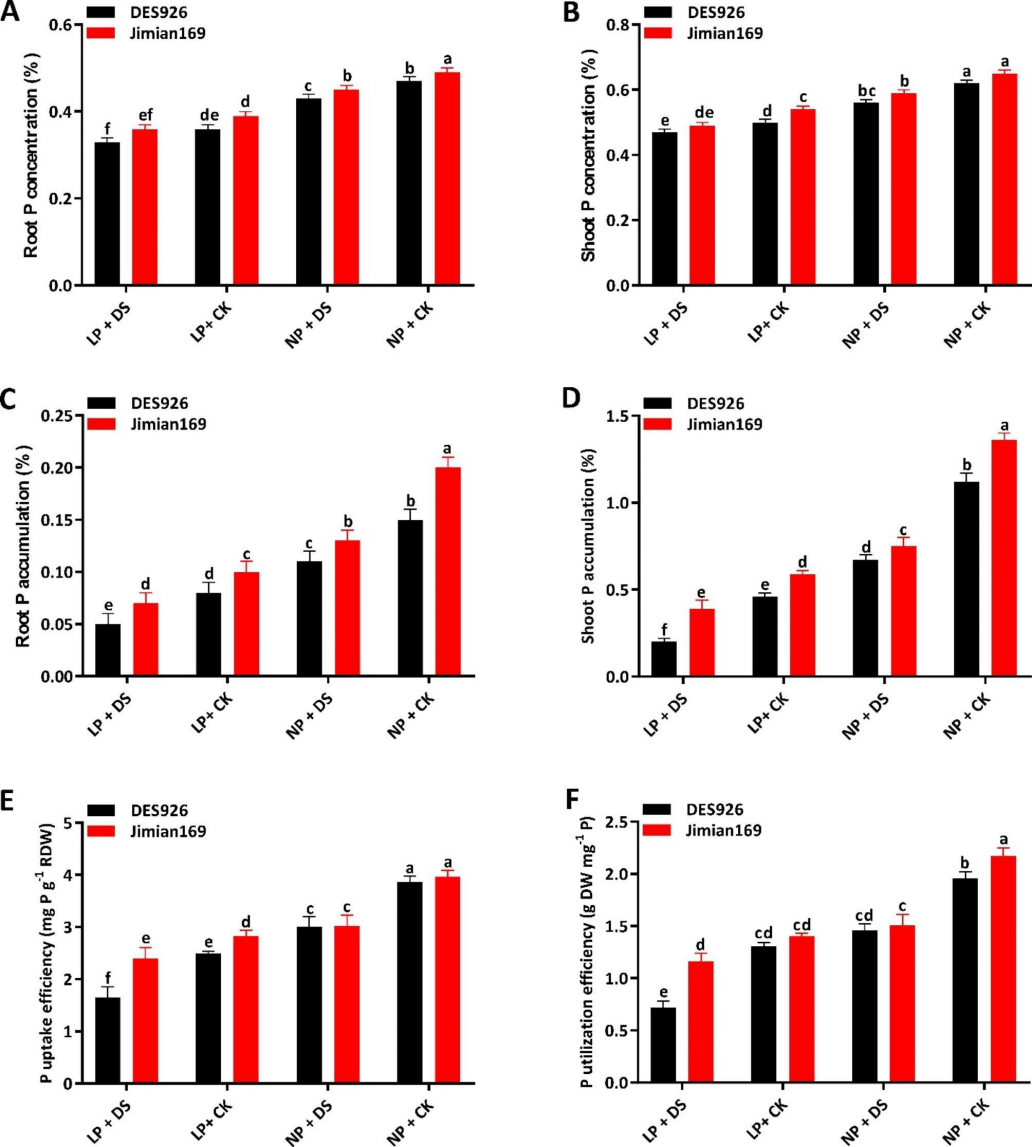
(**A**) Root P concentration (%), (**B**) shoot P concentration (%), (**C**) root P accumulation (%), (**D**) shoot P accumulation (%), (**E**) P uptake efficiency (mg P g^− 1^ RDW), and (**F**) P utilization efficiency (g DW mg^− 1^ P) of Jimian169 and DES926 under LP + DS (0.01 mM KH_2_PO_4_ + 10%PEG), LP + CK (0.01 mM KH_2_PO_4_ + 0%PEG), NP + DS (1 mM KH_2_PO_4_ + 10%PEG), NP + CK (1 mM KH_2_PO_4_ + 0%PEG).

The root and shoot P concentration abruptly dropped under drought stress with an approximate reduction of 11.4% and 7.9% under low P, while 8.7%, and 9.2% under normal P, respectively. In comparison with DES926, the root and shoot P concentration of Jimian169 increased by 6.6% and 5.2%, respectively (Fig. 1A, B). Compared to the control, a significant reduction in the root (34.1% and 30.0%) and shoot (44.1% and 42.7%) P accumulation was observed under low and normal P conditions. Between the genotypes, root, and shoot P accumulation was increased in Jimian169 by 21.2% and 20.7%, respectively (Fig. 1C, D). Similarly, drought stress reduced P uptake efficiency (PUpE) and P utilization efficiency (PUtE) by 23.8% and 30.7% under low P and 23.1% and 28.2% under normal P as compared to control. In comparison with DES926, PUpE and PUtE of Jimian169 increased by 8.3% and 12.7%, respectively (Fig. 1E, F).

### Malondialdehyde content and reactive oxygen species

**Fig. 2:**
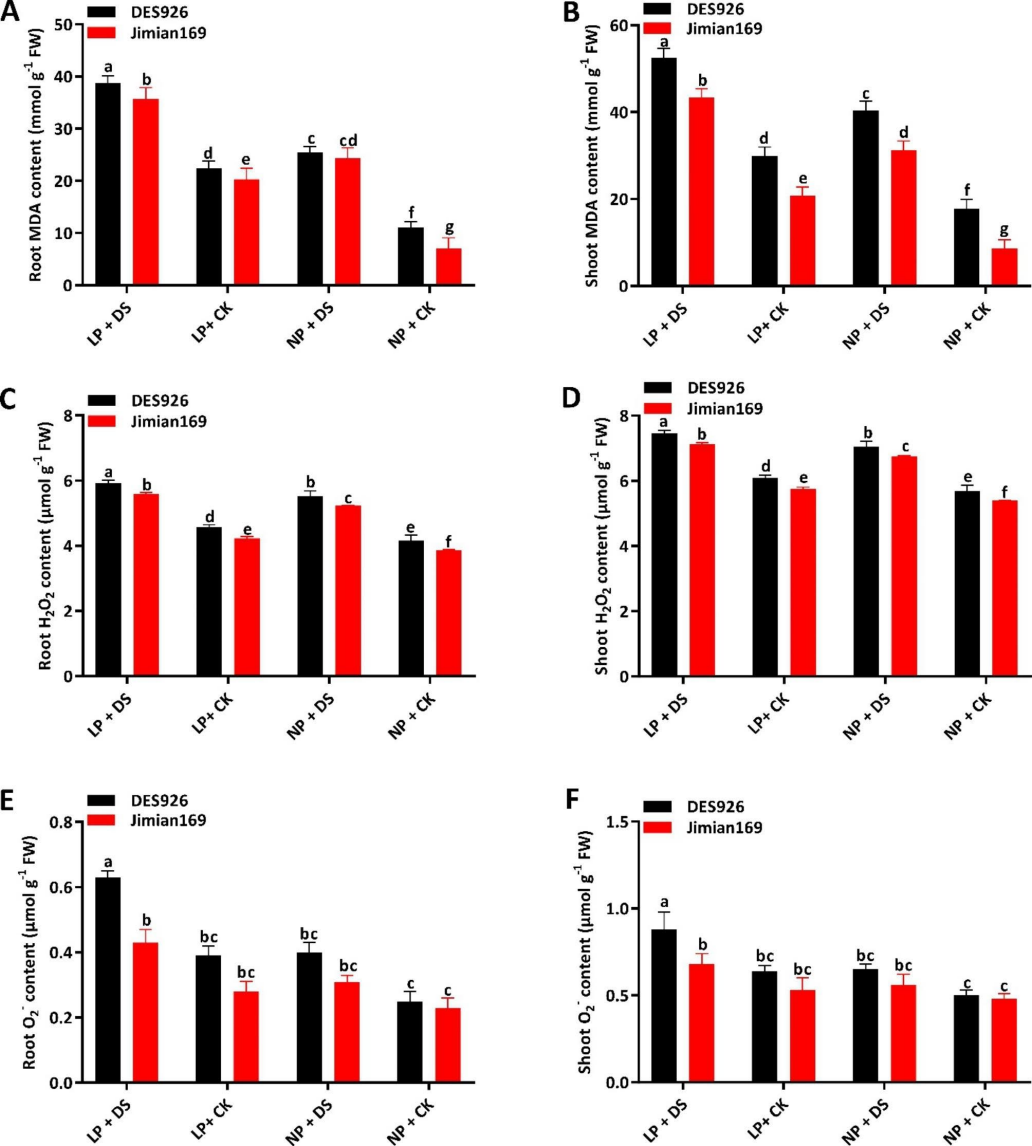
(**A**) Root malondialdehyde content (MDA; mmol g^− 1^ FW), (**B**) shoot malondialdehyde content (MDA; mmol g^− 1^ FW) (**C**) root hydrogen peroxide (H_2_O_2_; µmol g^− 1^ FW), (**D**) shoot hydrogen peroxide (H_2_O_2_; µmol g^− 1^ FW), (**E**) root superoxide anion (O_2_^−^; µmol g^− 1^ FW), (**F**) shoot superoxide anion (O_2_^−^; µmol g^− 1^ FW) of Jimian169 and DES926 under LP + DS (0.01 mM KH_2_PO_4_ + 10%PEG), LP + CK (0.01 mM KH_2_PO_4_ + 0%PEG), NP + DS (1 mM KH_2_PO_4_ + 10%PEG), NP + CK (1 mM KH_2_PO_4_ + 0%PEG).

Malondialdehyde (MDA) content was greatly induced by drought stress in the roots (55.3% and 146.3%) and shoots (33.8% and 91.7%) of both cotton genotypes under low and normal P conditions (Fig. 2A, B). Irrespective of the treatments, the MDA content was significantly higher in the roots (16.4%) and shoots (35.4%) of DES926 as compared to Jimian169 (Fig. 2A, B). As shown in Fig. 2, drought stress significantly increased ROS accumulation in the roots and shoots of cotton genotypes under both low and normal P conditions. Compared to the control, the H_2_O_2_ increased by 30.9% and 33.9% in the roots and 22.9% and 24.5% in the shoots under low and normal P conditions, respectively. Between the genotypes, DES926 had 6.7% and 5.1% higher H_2_O_2_ in the roots and shoots than Jimian169, respectively (Fig. 2C, D). Similarly, drought stress increased the level of O_2_^−^ in the roots (58.0% and 46.5%) and shoots (33.2% and 22.8%) under both low and normal P conditions. Regardless of the treatments, DES926 had significantly higher O_2_^−^ levels in the roots (32.9%) and shoots (18.3%) as compared to Jimain169 (Fig. 2E, F).

### Antioxidant enzymatic activities

**Fig. 3:**
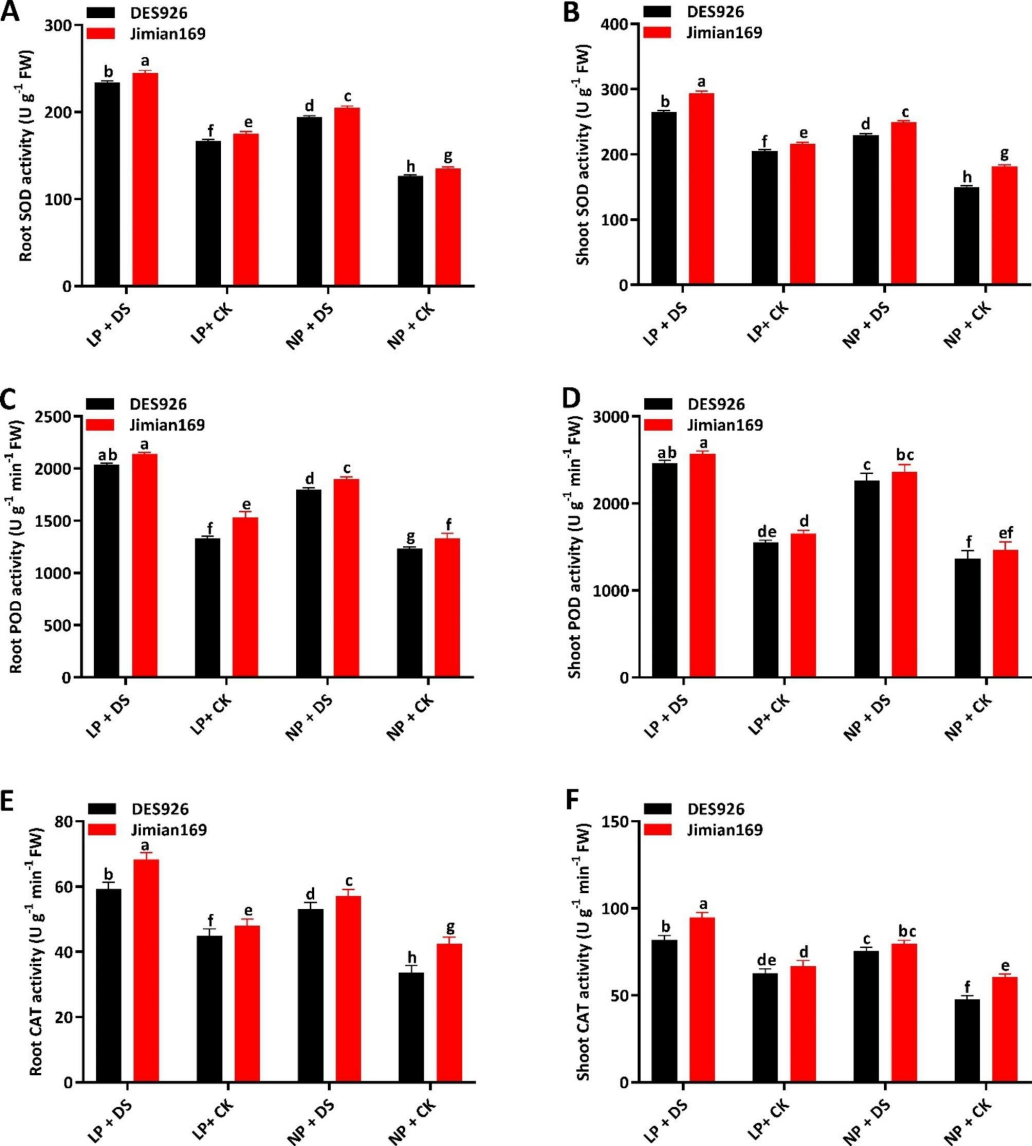
(**A**) Root superoxide dismutase activity (SOD; U g^− 1^ FW), (**B**) shoot superoxide dismutase activity (SOD; U g^− 1^ FW), (**C**) root peroxidase activity (POD; U g^− 1^ min^− 1^ FW), (**D**) shoot peroxidase activity (POD; U g^− 1^ min^− 1^ FW), (**E**) root catalase activity (CAT; U g^− 1^ min^− 1^ FW) (**F**) shoot catalase activity (CAT; U g^− 1^ min^− 1^ FW) of Jimian169 and DES926 under LP + DS (0.01 mM KH_2_PO_4_ + 10%PEG), LP + CK (0.01 mM KH_2_PO_4_ + 0%PEG), NP + DS (1 mM KH_2_PO_4_ + 10%PEG), NP + CK (1 mM KH_2_PO_4_ + 0%PEG).

Antioxidant enzymes like superoxide dismutase (SOD), peroxidase (POD), and catalase (CAT) were also studied to understand the tolerance of cotton genotypes against drought stress under low and normal P conditions (Fig. 3). The results showed that the activities of root SOD (23.4% and 16.7%), shoot SOD (24.9% and 19.7%), root POD (7.2% and 5.0%), shoot POD (6.5% and 4.1%), root CAT (22.8% and 17.6%), and shoot CAT (21.7% and 17.0%) were greatly induced by drought stress under low and normal P conditions as compared to control (Fig. 3). In comparison with DES926, the activities of root SOD, shoot SOD, root POD, shoot POD, root CAT, and shoot CAT in Jimian169 were higher by 5.1%, 12.8%, 9.8%, 9.5%, 16.2%, and 16.4%, respectively (Fig. 3).

### Free amino acid, total soluble protein, and total soluble sugar contents

**Fig. 4:**
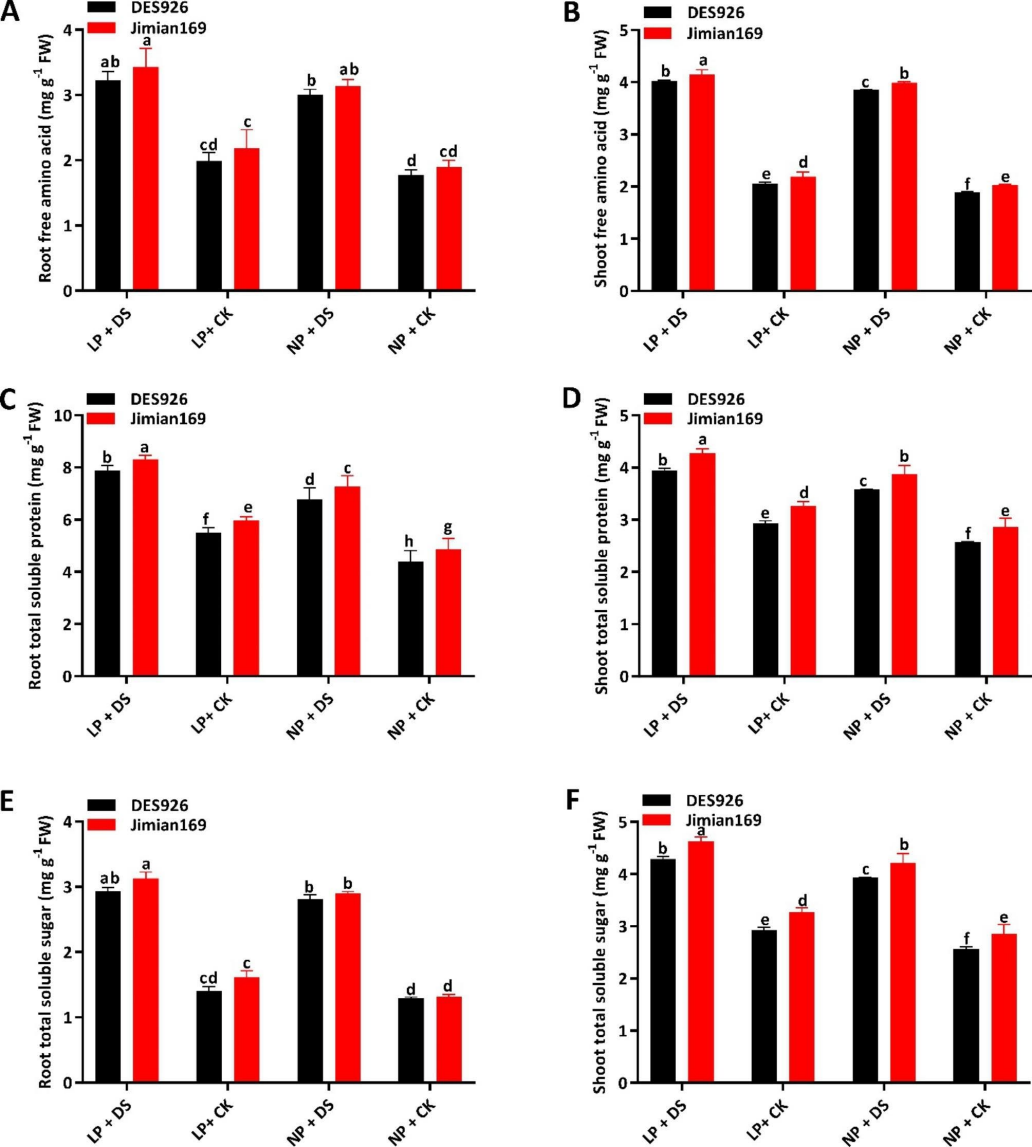
Root free amino acid (mg g^− 1^ FW), (**B**) shoot free amino acid (mg g^− 1^ FW), (**C**) root total soluble protein (mg g^− 1^ FW), (**D**) shoot total soluble protein (mg g^− 1^ FW), (**E**) root total soluble sugar (mg g^− 1^ FW), (**F**) shoot total soluble sugar (mg g^− 1^ FW) of Jimian169 and DES926 under LP + DS (0.01 mM KH_2_PO_4_ + 10%PEG), LP + CK (0.01 mM KH_2_PO_4_ + 0%PEG), NP + DS (1 mM KH_2_PO_4_ + 10%PEG), NP + CK (1 mM KH_2_PO_4_ + 0%PEG).

The osmoprotectants like free amino acids, total soluble protein, total soluble sugar, and proline content were also studied. The results showed that drought stress greatly improved the root-free amino acids (40.3% and 37.2%) and shoot-free amino acids (50.0% and 48.0%) under low and normal P conditions. The genotype Jimian169 had significantly higher root-free amino acids (6.1%) and shoot-free amino acids (4.3%) than DES926 (Fig. 4A, B). Moreover, drought stress significantly increased root total soluble protein (41.9% and 51.6%), shoot total soluble protein (32.5% and 37.2%), root total soluble sugar (100.7% and 114.0%), and shoot total soluble sugar (43.8% and 50.1%) of cotton genotypes under both low and normal P conditions. Irrespective of the treatments, root total soluble protein, shoot total soluble protein, root total soluble sugar, and shoot total soluble sugar of Jimian169 were higher by 0.4%, 8.9%, 6.4%, and 8.5% than DES926, respectively (Fig. 4C-F). The results further showed that root proline content (13.3% and 10.1%) and shoot proline content (11.9% and 8.9%) were significantly improved by drought stress under low and normal P conditions. The genotype Jimian169 had significantly higher root proline content (4.0%) and shoot proline content (3.6%) than DES926 (Figure S1).

### Correlation analysis

**Fig. 5:**
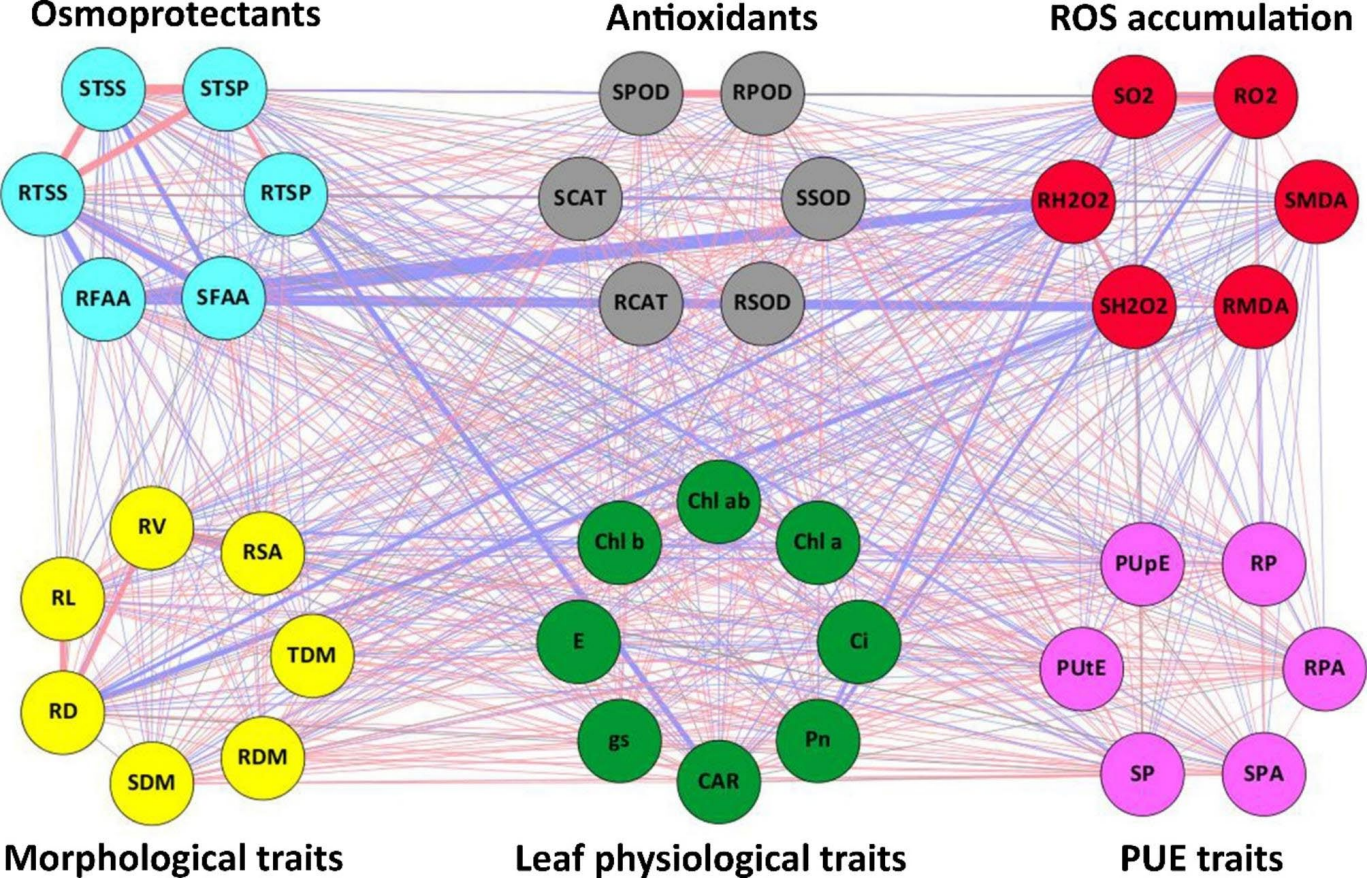
Relationships between morphological traits (yellow), leaf physiological traits (green), PUE traits (purple), ROS accumulation (red), antioxidants (gray), and osmoprotectants (water blue). Nodes shows the traits and edges (red positive and blue negative) represents the correlations. The thickness of the edges represents the strength of the correlation coefficient for each pair. SL; shoot length, RDM; root dry matter, SDM; shoot dry matter, TDM; total dry matter, RL; root length, RSA; root surface area, RD; root diameter, RV; root volume, Pn; photosynthetic rate, gs; stomatal conductance, E; transpiration rate, Ci; intercellular CO_2_ concentration, Chl a; chlorophyll a content, Chl b; chlorophyll b content, Chl ab; chlorophyll a + b, CAR; carotenoid contents, RP; root phosphorus concentrations, SP; shoot phosphorus concentration, RPA; root phosphorus accumulation, SPA; shoot phosphorus accumulation, PUpE; phosphorus uptake efficiency, PUtE; phosphorus utilization efficiency, RMDA; root malondialdehyde content, SMDA; shoot malondialdehyde content, RO2; root superoxide anion, SO2; shoot superoxide anion, RH2O2; root hydrogen peroxide, SH2O2; shoot hydrogen peroxide, RSOD; root superoxide dismutase, SSOD, shoot superoxide dismutase, RPOD; root peroxidase, SPOD; shoot peroxidase, RCAT; root catalase, SCAT; shoot catalase, RFAA; root free amino acids, SFAA; shoot free amino acids, RTSP; root total soluble proteins, STSP; shoot total soluble proteins, RTSS; root total soluble sugars, and STSS; shoot total solubvle sugars

The Pearson Correlationcorrelation analysis was performed to reveal the relationship between plant morphology, leaf physiology, P use efficiency, ROS accumulation, antioxidants, and osmoprotectants (Fig. 5). The correlation analysis revealed that a total of 40 nodes (traits) were connected with 741 edges in the network. Out of the total direct correlation, root morphological traits and osmoprotectants like total soluble protein and total soluble sugar have a strong positive relationship with each other. However, root diameter and free amino acids have a strong negative correlation with reactive oxygen species, especially H_2_O_2_ (Fig. 5). Overall, ROS accumulation has a negative correlation with other studied traits, suggesting that ROS accumulation induced by drought stress inhibits plant morphology, leaf physiology, and PUE, however, osmoprotectants and antioxidant enzymes greatly reduced the ROS accumulation and increased drought tolerance in cotton seedlings.

## Discussion

### Phosphorus improves morphophysiological tolerance of cotton under drought stress

Drought stress is one of the major abiotic stresses that affect plant growth throughout the world, particularly in arid and semiarid regions. Both short and long-term drought stress restricts plant growth and development and also hampers nutrient uptake more than any other abiotic stress [46]. In this study, various morphological variations were noticed in cotton seedlings exposed to PEG-induced drought stress, especially under low P condition (Table 1). The results showed that dry matter and growth of cotton genotypes were significantly decreased under drought stress than in the control condition (Table 1). We hypothesized that the reduction in growth under drought may be due to a decrease in chlorophyll contents, photosynthesis, and related traits, which result that ultimately produce low carbohydrates and dry matter. In line with these results, previous reports suggested that drought stress drastically reduced growth and various physiological processes like photosynthesis and related traits [47, 48]. Under drought stress, the significant reduction of these physiological traits led to several morphological variations like reduction in shoot length, dry matter, root architecture, etc. [49]. This negative response of various morphological and physiological traits under drought conditions is responsible for different performances of biomass and photosynthesis. The positive impact of the interaction of P and drought stress on photosynthesis suggests that P is important for regulating photosynthesis under drought stress. Therefore, normal P application significantly increased the seedling’s growth and alleviate the negative impact of drought stress. Similarly, P application significantly improved the growth of *Phoebe zhennan* under drought stress, indicating that the normal availability of P anchor the plants to survive the drought stress condition [11].

In addition, the reduction in dry matter is attributed to the decrease in leaf expansion, as the cells lose turgidity due to drought and subsequently reduced the photoassimilates for the development of new cells [50]. Though, some plants can decrease leaf size to restrict water loss through transpiration or improve their root water absorption. Therefore, a reduction in growth and biomass in cotton seedlings might be a drought-avoiding strategy of cotton genotypes followed by a reduction in photosynthesis as noted (Table 2). In addition to this, the reduction in growth and biomass under drought stress might be due to water deficiency that restricts water flow from roots to xylem and adjacent cells which decreases the availability of nutrients [51, 52]. These diverse and uneven responses of various plant species against P application in terms of growth and biomass are not so surprising and species identity, soil properties, nutrient availability, their interactions, and other factors control how the individual plants respond to an increased level of a specific nutrient.

The tolerance of drought stress in plants is governed by their ability to water retention [53]. Transpiration is the main process of water loss from plants. Under drought stress, plants reduce water loss by closing their stomata. Therefore, stomatal conductance and transpiration were significantly reduced [54, 55] which negatively affected photosynthesis. Reduction in stomatal conductance may be a strategy of cotton to avoid drought stress, allowing it to decrease transpiration to tolerate drought-induced stress. This stomatal closure and poor stomatal conductance are due to drought-induced synthesis of abscisic acid (ABA) in the roots followed by its transport to the leaves. The reduction of stomatal conductance is the result of low turgor pressure due to the K^+^ ion efflux induced by ABA [56]. This poor stomatal conductance restricts intercellular CO_2_ concentration and results in a reduction in photosynthesis as shown in Table 2 followed by a significant reduction in Rubisco activity [57]. Interestingly, P application greatly improved the photosynthesis of drought-stressed plants without affecting stomatal conductance and intercellular CO_2_ concentration. Similarly, previous reports also suggested that P application significantly improved photosynthesis under drought conditions [26, 58]. Thus, it is suggested that the application of P might be beneficial for cotton seedlings to tolerate drought stress.

### Phosphorus-efficient cotton genotype can tolerate drought stress through high antioxidant enzymatic activities and osmotic adjustment

Plants under stress conditions accumulated MDA which badly damage the cell membrane [59]. Therefore, MDA is an appropriate trait for the measurement of membrane lipid peroxidation. Under drought condition, we observed high MDA content and activities of antioxidant enzymes (Figs. 2 and 3). Similarly, a previous study also found higher enzymatic activities under drought stress conditions [60]. The high MDA content in drought-stressed plants was due to a significant increase in ROS production. Previously, it was reported that drought stress interrupts the equilibrium of ROS accumulation and utilization [61]. The accumulation of ROS enhances the photooxidative damage of photosynthesis and cell membrane peroxidation [62]. Thus to avoid photosynthetic damage in plants, maintenance of ROS is very important for normal photosynthesis [63] and redox equilibrium in the cell [64]. To maintain ROS equilibrium, plants have evolved several strategies of which the most important is the antioxidant system [65]. In the current study, cotton seedlings, especially Jimian169 showed strong resistance to ameliorating the damage induced by oxidative stress (Fig. 3). The higher antioxidant enzymatic activities and lower MDA content and ROS accumulation under normal P suggest an increase in the redox defense system of cotton Jimian169 against drought stress. Similarly, the application of nitrogen significantly increased the activities of SOD, POD, CAT, and soluble protein in maize leaves under drought stress [53, 66]. Thus drought stress regulates the antioxidant enzymatic activities in plants [53], however, its capacity mainly depends on P application.

ROS acts as signaling molecules in plant cells, however, their higher production can induce DNA fragmentation, protein degradation, lipid peroxidation, and ultimately cell death [14]. As shown in the results, the level of ROS was significantly higher in drought-stressed plants as compared to the control. However, ROS and MDA were further increased under combined low P and drought stress, indicating that the application of P inhibits ROS production under drought stress. Under drought stress, the increase in ROS level might be attributed to the reduction in photosynthesis (Table 2) which are in line with the results of the previous study [67]. To counter this, plants induce an antioxidant system as a defense mechanism against drought stress that helps to alleviate the oxidative stress and the subsequent cell damage from it. Moreover, the activities of antioxidant enzymes were higher in Jimian169 under drought conditions. Thus Jimian169 under drought stress greatly enhances the antioxidant defense system to overcome the excess ROS production and MDA level as compared to drought-stressed DES926 plants. In short, these findings suggest that the antioxidant defense system of DES926 is weak under drought stress, however, Jimian169 strengthened the efficiency of the antioxidant system to alleviate the negative impacts of drought.

In order to tolerate drought stress, plants mainly accumulate various osmolytes in the cytosol to improve the osmotic potential that subsequently improves the turgidity and maintains plant physiological and growth processes [68]. PEG-induced drought stress also alters the osmoprotectants such as free amino acids, total soluble protein, and total soluble sugars that regulate osmotic adjustment under drought stress [69]. Under drought stress conditions, cotton seedlings alter the levels of these osmoprotectants to maintain osmoregulation and are considered osmotic tolerance [70]. It was reported that alterations in free amino acids under drought stress reflect the accumulation of free amino acids [71], which acts as a stress indicator [72]. Among various osmoprotectants, amino acids are the most important osmo regulators that well maintain cell turgidity and prevent dehydration [73]. In this study, the higher levels of free amino acids and proline content in Jimian169 tissues regulate the osmotic adjustment and contribute to reducing osmotic potential and maintaining cell turgidity. Therefore, this study suggests that Jimian169 can maintain high levels of free amino acids and as a result improve drought stress tolerance. Additionally, soluble proteins and soluble sugars are important cellular components acting as a catalyst to improve stress tolerance in plants [70, 74]. Several researchers have confirmed the accumulation of osmoprotectants in different plants under stress conditions [37, 75–77]. Similarly, we have concluded that Jimian169 seedlings under drought stress had significantly improved the production and accumulation of osmoprotectants that serve as an osmotic potential and maintain cell turgidity thus avoiding dehydration of the tissues.

## Conclusions

In summary, PEG-induced drought stress significantly affected the growth and metabolism of both cotton genotypes as shown by the reduction in dry matter, photosynthesis, chlorophyll traits, PUE, antioxidant enzymatic activities, and the increase in MDA level, especially in DES926. However, the higher osmolytes accumulation greatly maintained the tissue osmotic potential, suggesting its significance in improving drought stress tolerance. Moreover, Jimian169 has significantly higher antioxidant enzymatic activities, accumulation of osmoprotectants, PUE, and reduced the MDA level; hence its photosynthetic rate was improved. Thus, it is of great interest to study the underlying mechanism of stress tolerance in Jimian169 that will help in future breeding for combined low P and drought stress tolerance.

## Electronic supplementary material

Below is the link to the electronic supplementary material.

Supplementary Material 1

## Data Availability

All data generated or analyzed during this study are included in this published article.
