# Supplementary material for: Differential responses of contrasting low phosphorus tolerant cotton genotypes under low phosphorus and drought stress
Source: BMC Plant Biol. 2023 Mar 30;23:168. doi: 10.1186/s12870-023-04171-5 (PMC10061777; doi:10.1186/s12870-023-04171-5)
Supplement: Supplementary file 1 — Supplementary Material 1 [file 12870_2023_4171_MOESM1_ESM.docx]

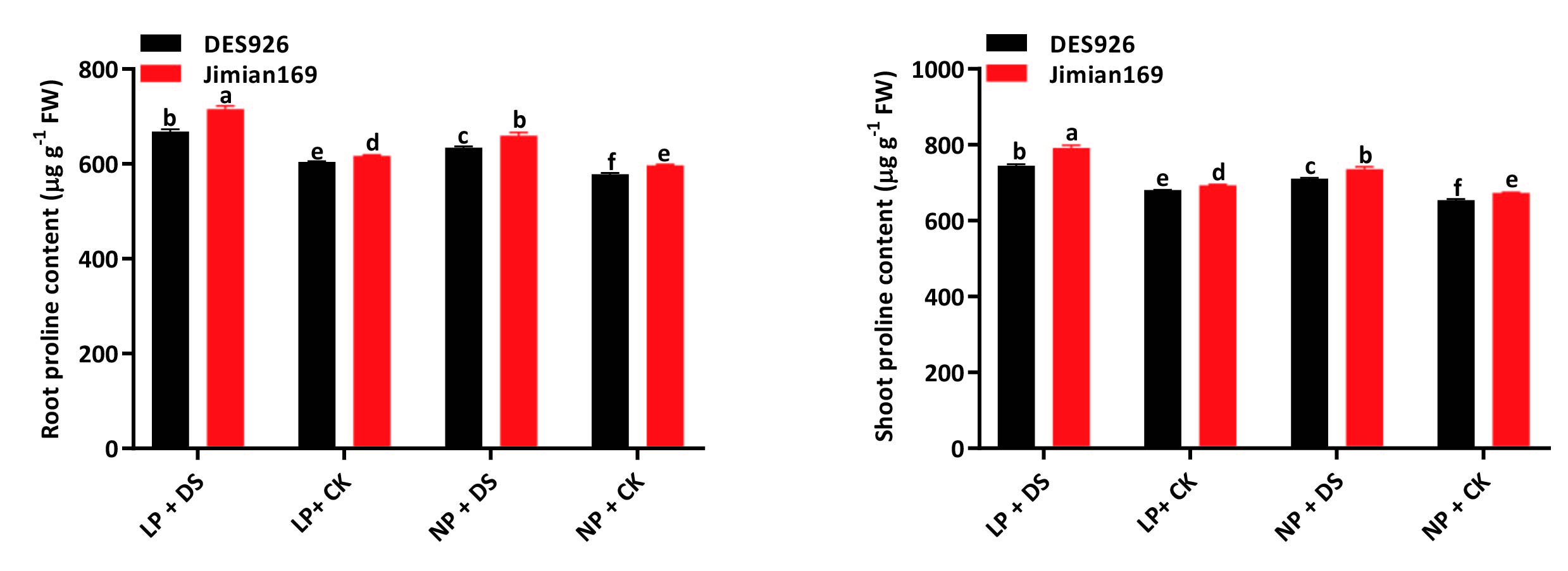


Figure S1. (A) Root proline content (µg g^-1^ FW) and (B) shoot proline content (µg g^-1^ FW) of Jimian169 and DES926 under LP + DS (0.01 mM KH_2_PO_4_ + 10%PEG), LP + CK (0.01 mM KH_2_PO_4_ + 0%PEG), NP + DS (1 mM KH_2_PO_4_ + 10%PEG), NP + CK (1 mM KH_2_PO_4_ + 0%PEG).
